# Restoring Finger-Specific Sensory Feedback for Transradial Amputees via Non-Invasive Evoked Tactile Sensation

**DOI:** 10.1109/OJEMB.2020.2981566

**Published:** 2020-03-19

**Authors:** Manzhao Hao, Chih-Hong Chou, Jie Zhang, Fei Yang, Chunyan Cao, Pengyu Yin, Wenyuan Liang, Chuanxin M. Niu, Ning Lan

**Affiliations:** ^1^ Institute of Medical RoboticsShanghai Jiao Tong University 12474 Shanghai 200240 China; ^2^ Laboratory of NeuroRehabilitation EngineeringSchool of Biomedical EngineeringShanghai Jiao Tong University 12474 Shanghai 200030 China; ^3^ Department of Functional NeurosurgeryRuijin Hospital, School of MedicineShanghai Jiao Tong University 12474 Shanghai 200025 China; ^4^ National Research Center for Rehabilitation Technical Aids Beijing 100176 China; ^5^ Department of Rehabilitation MedicineRuijin Hospital, School of MedicineShanghai Jiao Tong University 12474 Shanghai 200025 China

**Keywords:** Evoked tactile sensation (ETS), magnetoencephalography (MEG), prosthetic hand, sensory feedback, transcutaneous electrical nerve stimulation (TENS)

## Abstract

*Objective:* This study assessed the feasibility to restore finger-specific sensory feedback in transradial amputees with electrical stimulation of evoked tactile sensation (ETS). *Methods:* Here we investigated primary somatosensory cortical (SI) responses of ETS using Magnetoencephalography. *Results:* SI activations revealed a causal correlation with peripheral stimulation of projected finger regions on the stump skin. Peak latency was accountable to neural transmission from periphery to SI. Peak intensity of SI response was proportional to the strength of peripheral stimulation, manifesting a direct neural pathway from skin receptors to SI neurons. Active regions in SI at the amputated side were consistent to the finger/hand map of homunculus, forming a mirror imaging to that of the contralateral hand. With sensory feedback, amputees can recognize a pressure at prosthetic fingers as that at the homonymous lost fingers. *Conclusions:* Results confirmed that the direct neural pathway from periphery to SI allows effective communication of finger-specific sensory information to these amputees.

## I. Introduction

Seamless flow of neural information between motor efferent and sensory afferent is a prerequisite for dexterous control of human hand manipulation [1]. When operating prosthetic hands, missing afferent information back to the brain of amputees undermines their ability to achieve effective control of motor functions, resulting in rejection by many amputees who wear prosthetic hands [2], [3]. Therefore, providing sensory afferent information to the brain of amputees becomes one of the frontiers of research and application in both sensorimotor science and neurorehabilitation engineering [4]–[14].

Various neural techniques have been developed and evaluated to provide sensory feedback for prosthetic hands. Non-invasive methods, such as vibrotactile [15], [16] and electrotactile using TENS [17]–[19], are more readily applied to supply a substitutional sense of awareness for the operation of prosthetic hands [17]–[19]. More natural sensory feedback often entails direct access of sensory nerve fibers in the ascending pathway or in the somatosensory cortex (SI) via invasive neural interface technologies [20]. For example, sensory effects can be obtained with peripheral nerve stimulation techniques [12], [21]–[24], and with direct stimulation of SI neurons [7], [25], [26]. Peripheral nerve stimulation using nerve cuff electrodes [12] have demonstrated relatively long-term stability and functional improvement in prosthetic hands [27]. Cortical stimulation has also produced finger-specific sensations [7] with rich sensory modalities [25], [26] in patients with braingate implants. These advances of sensory feedback techniques present a variety of choices for patients of various conditions.

The quality of sensory effects depends on the richness of sensory information communicated with a neural interface technology. Sensory information conveyed via vibrotactile or electrotactile techniques is often substitutional, and restricted to a single degree of freedom for force or position [28]–[31]. Somatotopic information may be encoded through multiple sites of electrotactile stimulation [32]. However, the adaption to non-homologous information may be contingent on the plasticity of central sensory nervous system [33], [34]. Recapturing somatotopic sensation usually requires stimulation of sensory fibers innervating specific parts of the body at peripheral nerves [12], [21]–[24] or at sensory SI cortex in the brain [7], [25], [26]. We demonstrated in our previous study that the neural interface technique based on evoked tactile sensation (ETS) may have the potential to produce finger-specific sensory effects [35], [36]. In this paper, we further developed this technique, substantiated its capacity, and clarified its neural basis for restoring finger-specific sensations.

In some transradial amputees, a unique phenomenon exists in their stump skin, that is the lost fingers can be felt when stimulating specific regions of stump skin either mechanically [37], [38] or electrically [35]. This phenomenon was initially explored as a plausible way to provide sensory feedback with mechanovibrators [4]. In our previous work, we evaluated this phenomenon further using electrical stimulation via surface electrodes placed over the targeted skin regions [39]. We found that transcutaneous electrical nerve stimulation (TENS) produced six types of sensations, corresponding to the different kinds of sensory receptors in the skin, including light touch, pressure, vibration, buzz, numbness and tingling pain. And specifically, subjects with this phenomenon reported that sensation was felt at a part of the fingers of the amputated hand, for example, at the fingertip, other phalanges of the fingers, or the dorsal hand. Different sensory effects occurred orderly with modulation of stimulation amplitude, or pulse width, or frequency. These modalities of sense were similar to those experienced at the normal skin with TENS [35], [40]. To differentiate this phenomenon with phantom limb sensation (PLS) experienced by some amputees [41], we coined this phenomenon as evoked tactile sensation (ETS), since it requires an external stimulus to elicit at specific regions of the stump skin.

Here in this paper, we evaluated further the non-invasive ETS technique as a way to supply finger-specific natural sensory information from prosthetic hands to the sensory system of amputees. We explored the neural mechanism of ETS using neural imaging technique of Magnetoencephalography (MEG), and investigated the evoked responses at the somatosensory cortex (SI) [42]. This paper reports the main findings that the central responses elicited with ETS corroborated the subjective feelings reported by amputees. This suggested a direct neural pathway that can carry finger-specific information from the peripheral stump skin to the somatosensory cortex of these amputees. Experimental results reaffirmed the feasibility to use ETS as a non-invasive neural interface to restore a high fidelity, finger-specific sensory feedback pathway for prosthetic hands using simultaneous multi-channel sensory stimulation.

## II. Results

### A. Magnetoencephalography (MEG) Responses in the Somatosensory Cortex (SI) During Stimulating the Projected Finger Map (PFM) Regions of Stump, As Well as Fingers of the Contralateral Hand

**Figure 1.**
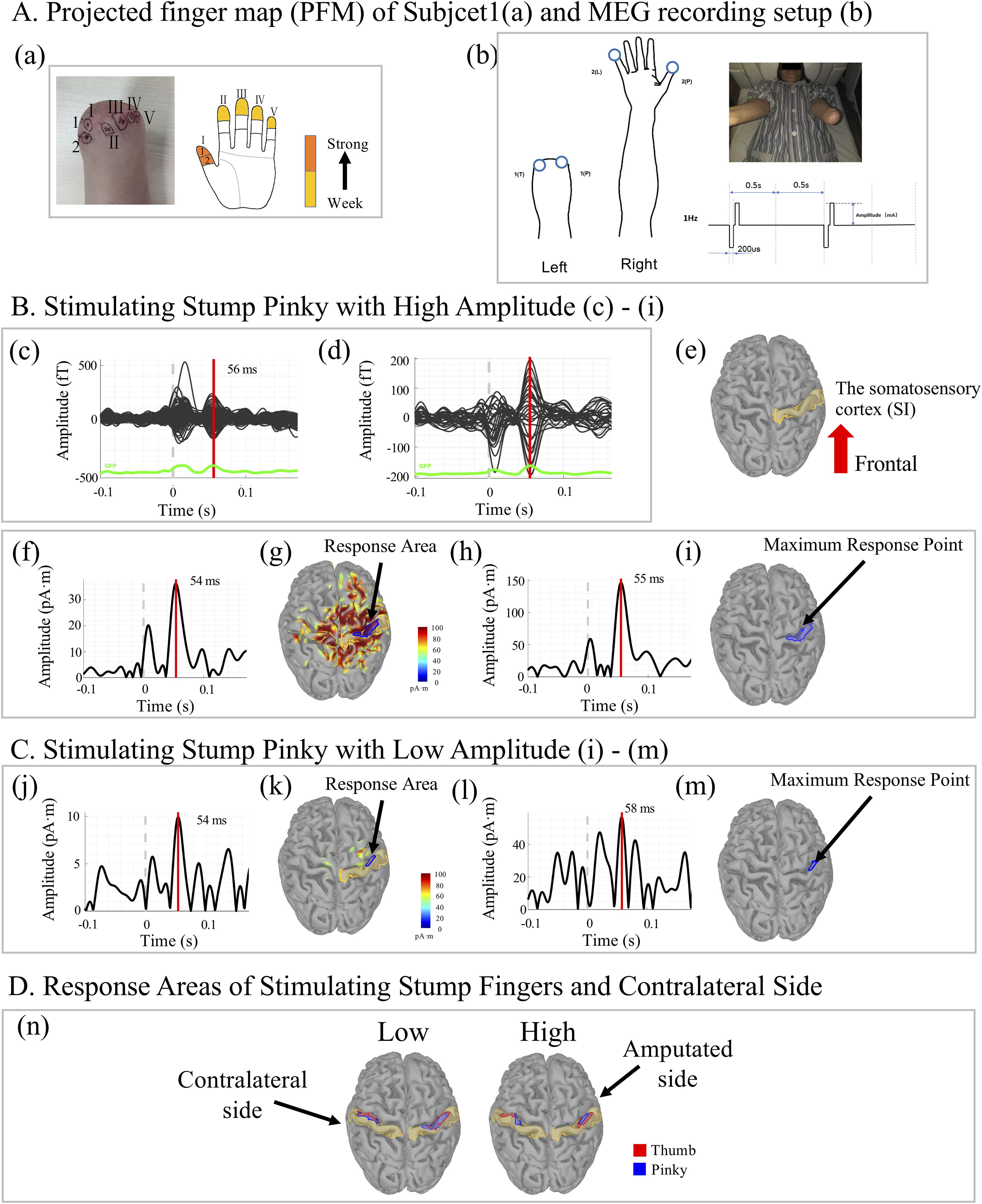
Somatosensory cortex (SI) responses of evoked tactile sensations (ETS) recorded by magnetoencephalography (MEG) in Subject 1. (a) The projected finger map (PFM) of Subject 1. (b) MEG recording was done in a shielded room, where electrical stimulations on the projected finger regions of the stump and the fingers of the contralateral normal hand were delivered with biphasic pulse trains of 1 Hz frequency. (c)-(i) Present a trial that the projected pinky region on the stump was stimulated with high current amplitude (11.25 mA with 2.5 cm diameter stimulation electrode). 102 channels of event related magnetic flux recordings of raw MEG in the whole brain and the right parietal lobe are shown in (c) and (d), respectively. The response time of magnetic flux is 56 ms. The activities seen at time=0 may be due to noise inputs. (e) illustrates the brain model of Subject 1 and the SI area is labelled in yellow. (f) Plots the time profile of the average current dipole of the SI area. The brain activities of response at peak time (54 ms) of SI are shown in (g). The blue circle labels the response area (RA). (h) Plots the time profile of the average current source densities in the RA. The response time (RT) is defined as the peak time of (h), which is 55 ms. The maximum response point is shown in (i), which is defined as the vertex having the max value in the RA at the moment of RT. (j)-(m) present a trial that projected pinky region on the stump is stimulated with low amplitude (3.75 mA with 2.5 cm diameter stimulation electrode). The response areas in SI of 4 sites (projected thumb region of the stump, projected pinky region of the stump, the contralateral thumb and pinky) with high and low are presented as (n).

**Figure 2.**
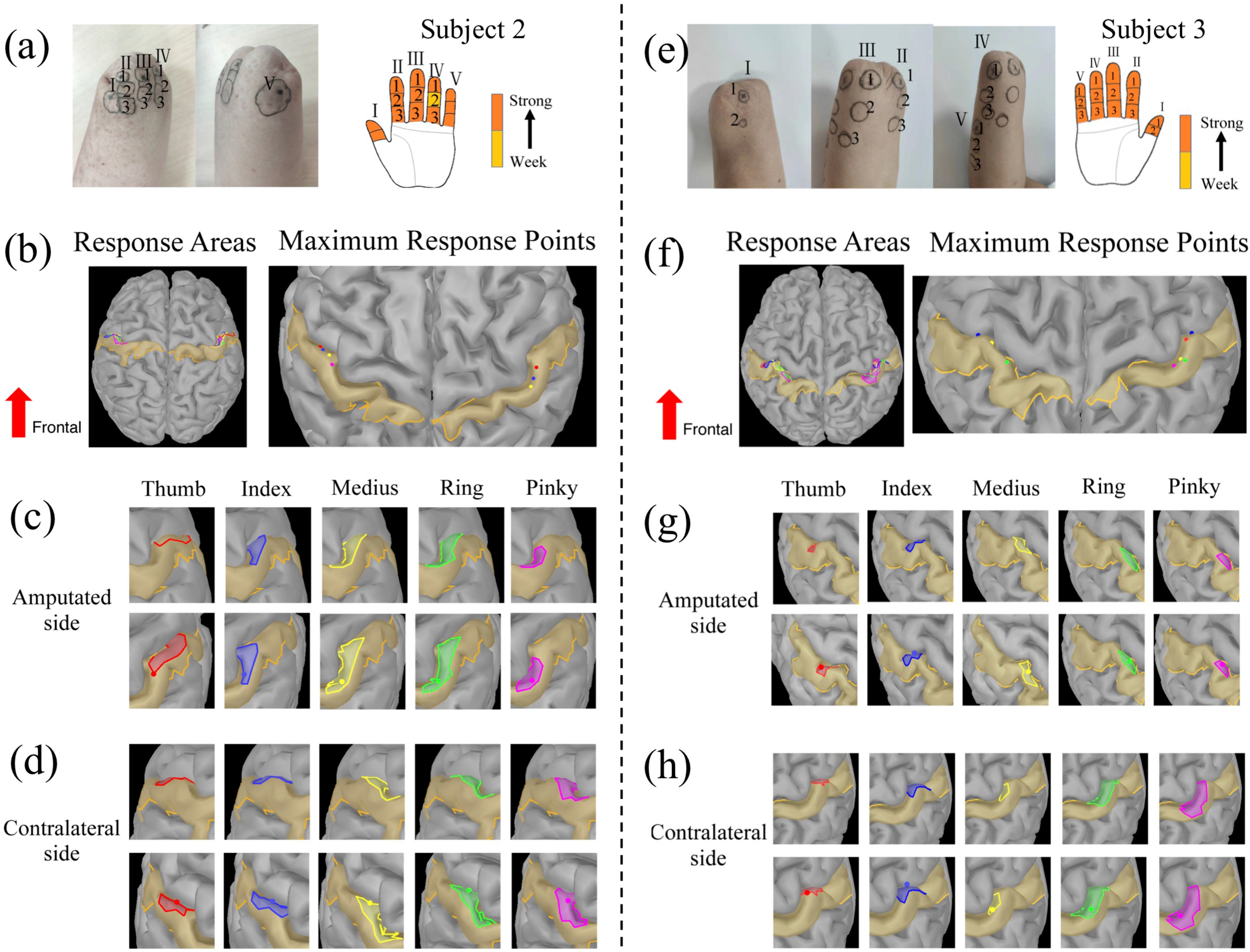
Response areas (RAs) and maximum response points (MRPs) of evoked tactile sensations (ETS) in the somatosensory cortex (SI) of Subject 2 (a-d) and Subject 3 (e-h). The PFMs of Subject 2 and Subject 3 are shown in (a) and (e), respectively. (b)-(d) Illustrate RAs and MRPs of SI when stimulating the five PFM regions on the stump and the five fingers of the contralateral hand of Subject 2. The overview of RAs and MRPs is presented in (b). (c) Depicts the SI responses in the amputated side. Pictures in the upper row show the RAs of five PFM regions from the same perspective; pictures in the lower raw show the position of the MRPs after fine-tuning of the pitch angle. (d) Exhibits the RAs and the MRPs of contralateral SI. (f)-(h) Illustrate the RAs and MRPs of SI for Subject 3 in the similar order.

We evaluated the SI response of evoked tactile sensations in Subject 1 initially. The projected finger map (PFM) regions of five fingers of Subject 1 are shown as Fig. 1(a). The distribution of PFM regions at the stump in general follows the order of thumb, index, middle, ring and pinky fingers from radial to ulnar nerve innervations (also see Fig. 2(a) & (e)).

We employed the neuroimaging technique of Magnetoencephalography (MEG) and Magnetic Resonance Imaging (MRI) to reconstruct central activities in the somatosensory cortex (SI) during electrically stimulating the thumb and pinky finger regions of the PFM in Subject 1. The thumb and pinky fingers of the contralateral hand were also stimulated as control comparison for SI responses. A train of bi-phasic pulses of 1 Hz was delivered to one region at a time, shown as Fig. 1(b). The subject was asked to confirm which finger was felt with stimulation before MEG recording. 102 channels of event related magnetic flux recordings of raw MEG in the whole brain and the parietal lobe are shown in Fig. 1(c) and 1(d), respectively. They represented the brain response when the pinky region of the PFM specifically was stimulated. The current source of the evoked response was computed using a software package with MRI scan of subject's head. Fig. 1(e) shows the SI area in the brain model of Subject 1. The time response of average current dipole of vertexes located in SI is shown in Fig. 1(f). There was a peak around 54 (ms) following the peripheral stimulus. This latency time was consistent with the time of sensory nerve conduction from the skin of transradial stump to the SI of the brain. The blue circle was labelled as the response region in SI observed with visual inspection, which represented roughly the response area (RA) in the SI when stimulating the pinky PFM. Fig. 1(h) plots the time profile of the averaged current dipole density in the RA. The response time (RT) was defined as the peak time of averaged current dipole density in Fig. 1(h), which was 55 (ms). Fig. 1(i) illustrates the maximum response point (MRP), which was identified at vertexes with the maximum value of current dipole at the instant of peak response time.

The response areas and maximum response points (MRP) in the SI with stimulating PFMs of the thumb and pinky finger and the contralateral hand are shown as Fig. 1(n). The response areas of thumb and pinky fingers of the contralateral hand in the SI are consistent with the homunculus mapping of hand, with the thumb response area more lateral to that of the pinky in SI. Stimulating the PFMs of thumb and pinky regions in the stump resulted in a similar distribution of MRPs to those of the contralateral hand in the SI.

MEG was sensitive to different levels of stimulation at the PFM. With stimulation of high and low intensities, the subject also felt distinguishable high and low levels of sensation. Fig. 1(j) to (m) depict a trial stimulating the pinky region of the PFM with low amplitude. It is clear that both the area and the peak value of the response area was smaller with low stimulation amplitude (3.75 mA with 2.5 cm diameter stimulation electrode), and larger with high stimulation amplitude (11.25 mA with 2.5 cm diameter stimulation electrode).

This observation indicates that stimulating PFM regions of the stump produced not only subjective feelings of the lost fingers being touched or pressed, but also the central SI responses consistent to the sensory neuroanatomical pathway and the organization of intact somatosensory cortex. This provides a direct evidence that the PFM regions of the skin on the stump of amputees are neuroanatomically connected to the cortical SI areas of the original fingers. One possibility for this newfound neural connection is that the severed sensory nerve fibers innervating the hand fingers regrow into the stump skin regions after amputation, thus forming the PFM of individual fingers. This hypothesis will be further discussed later the paper.

MEG response times (RT) obtained in 3 transradial amputee subjects when stimulating their projected finger regions in the stump skin and contralateral fingers were listed in Table I of supplementary materials. The RTs were in the similar order from 55 (ms) to 70 (ms) in all subjects for both amputated and contralateral sides.

### B. Landscapes of SI Response for Stimulating Five Finger Regions in the PFM and the Contralateral Hands

Another set of experiment was performed to compare the SI responses during stimulating five fingers of the PFM regions with those of the contralateral hand. The PFM regions of five fingers for Subject 2 and Subject 3 are shown as Fig. 2(a) and Fig. 2(e), respectively. The response areas and maximum response points in the SI with stimulation of five finger regions of the PFM and the contralateral hand are shown in Fig. 2(b) and Fig. 2(f), respectively. The five RAs from thumb to pinky fingers were consistent to the homunculus organization in both subjects. However, the RAs of adjacent fingers often overlapped with each other. The RAs of five contralateral fingers from thumb to pinky were located in a sequential order from lateral to medial regions in the SI. The RAs of the PFM from thumb to pinky were symmetrical to those of the contralateral hand. In Subject 2, the maximum response points of medius and ring finger of the contralateral hand appeared at almost the same location (Fig. 2(b) & (d)). Similarly, the maximum response points of the projected medius, ring and pinky fingers were very close to each other (Fig. 2(b) & (c)). For Subject 3, there was little overlap between the maximum response points of five contralateral fingers (Fig. 2(f) & (h)). However, the maximum response points of the projected ring and pinky fingers coincided, and those of the projected thumb and index fingers overlaid with each other (Fig. 2(f) & (g)).

These results corroborate the subjective feelings of lost fingers reported by subjects when their PFM areas were stimulated, with the SI responses revealed by MEG. Therefore, the finger-specific sensation is truly elicited by the external stimulus applied to the PFM.

### C. Correlation of the Intensity of SI Response With the Amplitude of Stimulus Delivered to Five Fingers in the PFM and the Contralateral Hand

**Figure 3.**
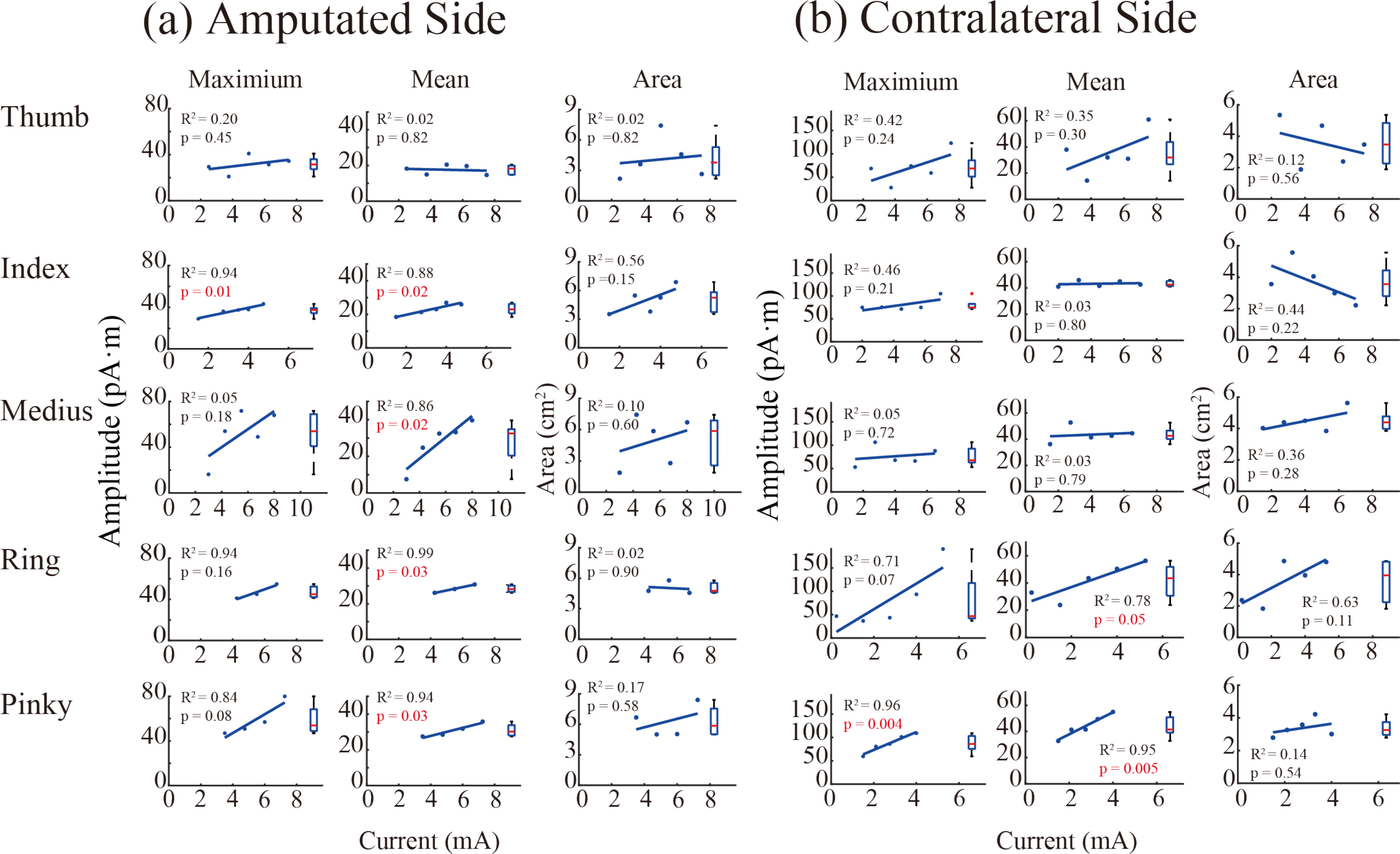
Causal analysis of intensities of SI response with varying amplitudes of peripheral stimulus in Subject 2, in which different amplitudes of stimulation current pulses were delivered to PFM regions of five fingers (a) and fingers of the contralateral hand (b). Three indices of SI responses were evaluated. The current dipole value of the maximum response point (MRP) at the response time (RT) moment is defined as the ‘Maximum’ value. The ‘Mean’ value is the peak value corresponding to the RT moment of the time profile of the average current dipole in the response area (RA). The ‘Area’ value is the actual area of SI responses. p-values that are less than or equal to 0.05 are labelled with red color.

A separate experiment was designed to show the causal correlation between central SI responses and external stimulations of varying intensities. The intensities of SI responses with different levels of stimulus of five fingers of the PFM and the contralateral hand of Subject 2 are shown in Fig. 3. Three to five distinguishable levels of stimulation amplitude were delivered to each stimulation sites at five fingers of the PFM and the contralateral hand. Three indices were examined to represent the central response intensity. The index of mean response intensity was defined as the peak value of the averaged current dipole densities in the response areas. The other two indices were values at the maximum response point within the response area, and the response area itself. These three indices may characterize the profile of central responses in SI. From Fig. 3, it is clear that for five fingers of both the contralateral hand and the PFM of the stump, the maximum intensity and mean intensity displayed a positive correlation to the amplitude of peripheral electrical stimulus in half cases (p-values of significance were labelled with red color). These imply a causal relationship between the central responses in SI and the external stimulus applied to the fingers of the PFM and contralateral hand.

### D. Subject's Sensing of the Pressure Applied to the Prosthetic Fingers by Way of Evoked Tactile Sensation

The conspicuous sensory modalities that the subject felt when stimulating the PFM regions were further investigated to provide a way of natural sensory feedback. Stimulus pulse width was varied while the amplitude and frequency were fixed in these tests. The modality of buzz sensation was chosen to encode the prosthetic pressure because of its wide modulation range. A stimulation frequency of 50 Hz was used throughout the experiment.

**Figure 4.**
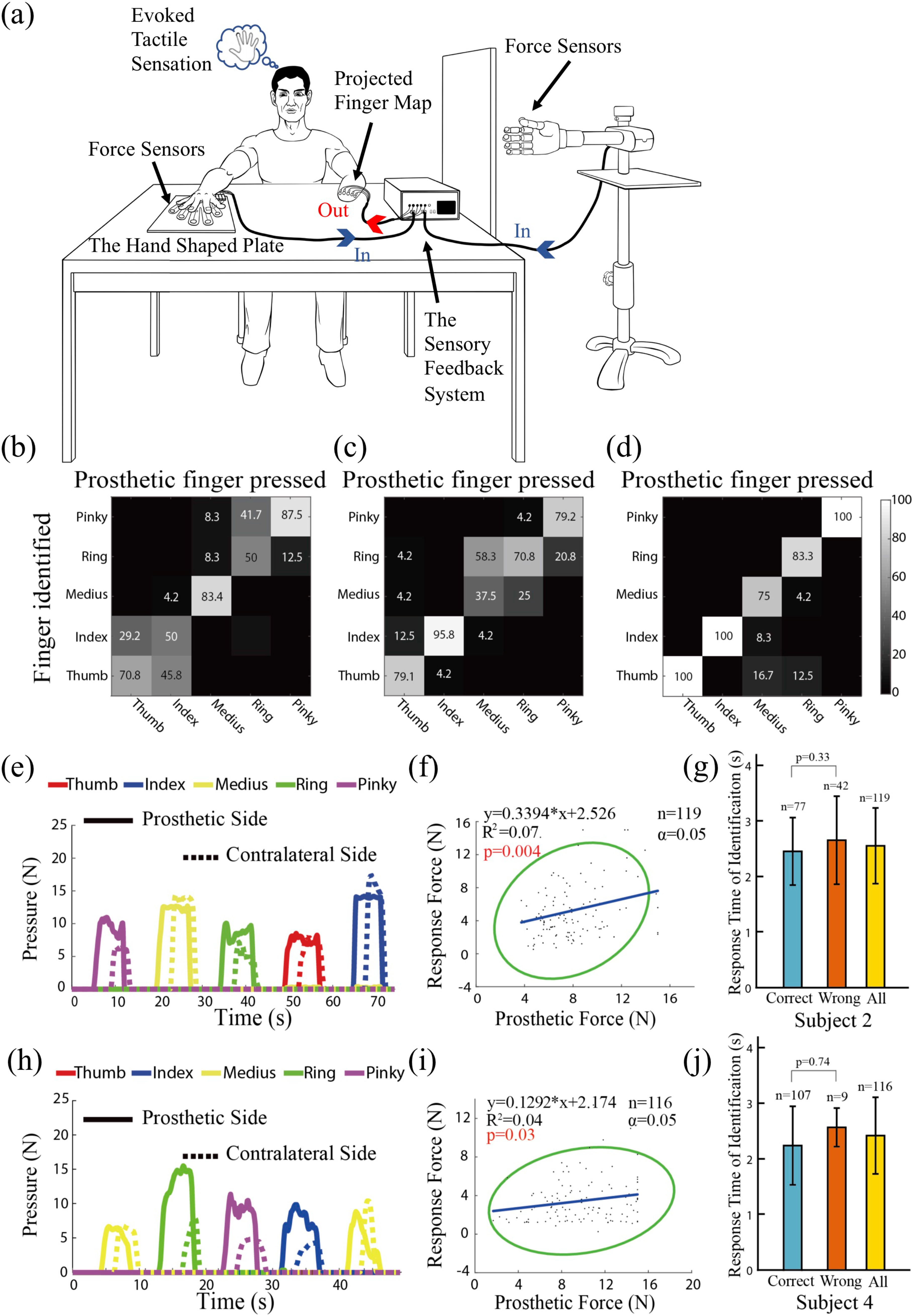
Results of the finger-to-finger identification experiment by Subject 2, Subject 3 and Subject 4. (a) Experimental setup, in which the subject sensed the pressure of a prosthetic finger by way of a multi-channel sensory feedback system, then pressed the sensors on a hand shaped plate using contralateral fingers. The experiment is demonstrated in Video 1 and Video 2. Confusion matrixes (b) - (d) present the accuracy of finger-to-finger identification of the three subjects. (e) and (h) depict response force of contralateral fingers by Subject 2 and Subject 4, respectively. The correlation between the response force and the prosthetic pressure force is illustrated in (f) and (i). The corresponding 95% confidence ellipses surrounded all paired force points whether the identification was correct or wrong. (g) and (j) show the response times with correct, or wrong identification, and all identifications.

To verify sensory restoration for the prosthetic hand via ETS, a real-time non-invasive tactile sensory feedback system was designed. This sensory feedback system could sample multiple channels of pressure information applied to the fingers of prosthetic hand, and encode the pressure information into multiple channels of electrical stimuli, which was delivered to the surface electrodes placed at the site of PFM on the stump. In the experiment, the subject was asked to press the corresponding contralateral finger that matched the prosthetic finger to which a pressure was applied. The force registered by the sensors of contralateral fingers could indicate which finger of the prosthetic hand and how hard the prosthetic finger was being pressed. The experiment set up is illustrated in Fig. 4(a) and exemplified in Video 2 provided in supplementary files.

Results obtained in three subjects are shown in Fig. 4(b) to (j). The confusion matrixes in Fig. 4(b) to (d) illustrate the rate of correct finger identification performed by Subject 2, Subject 3 and Subject 4, respectively. For Subject 2, the accuracy of identification for the thumb, middle and pinky fingers was above 70%, but it was about 50% for the index and ring fingers. For Subject 3, only the accuracy of identifying the middle finger was below 70%. In both subjects, most errors of identification occurred at the adjacent fingers, probably due to closely distributed projected finger regions in the stump. For Subject 4, the identification accuracy for the thumb, index and pinky was 100%. The lowest accuracy was 75% for the medius. Subject 4 has a clearly separated PFM for five fingers, see Fig. 1(c) in [35]. Fig. 4(e) & (h) present the time profiles of pressures recorded by force sensors at the prosthetic fingers and at the contralateral fingers of Subject 2 and Subject 4. In this experiment, the subjects were asked to press the force sensors using the contralateral fingers according to the perceived strength of electrical stimulation at the site of PFM, which was correlated to the pressure at prosthetic fingers. There was a clear latency time at the onset from prosthetic pressure to that of the contralateral pressure. But the subjects were able to stop pressing the contralateral fingers quickly at the drop of prosthetic pressure. The correlation between the prosthetic pressures and the response pressures was illustrated in Fig. 4(f) & (i), without discriminating whether the finger identification was correct or not. Although data points were scattered in a relatively large area, they still showed a positive trend of distribution (p < 0.05 in both cases). The average response time of identification for Subject 2 was 2.55+/−0.68 (s), and 2.41+/−0.69 (s) for Subject 4, shown as in Fig. 4(g) & (j). This result indicates that pressure information at the prosthetic fingers is transmitted to subjects via the non-invasive interface, perceived by subject's sensorimotor system, and intended motor commands were executed by their muscles of contralateral fingers. This further demonstrated that it is feasible to rebuild the interrupted sensory-motor information flow between prosthetic hands and amputees via evoked tactile sensation.

## III. Discussion

This study confirmed that seamless neural information flow from sensory afferent to motor efferent with finger specificity in amputees can be restored with evoked tactile sensation (ETS). This finding is significant in that ETS can re-establish finger-specific sensory ability for these amputees with non-invasive electrical stimulation. Furthermore, the sensory information can be interpreted naturally as that arising from the fingers of amputated hands. This feature may facilitate the perception of task performance by the amputee and planning of proper motor actions based on the sensory information received. These findings substantiated feasibility to implement non-invasive ETS based sensory feedback for closed-loop control of neuroprosthetic hands.

It was found that activation response areas in the somatosensory cortex (SI) during electrical stimulation of the peripheral PFM areas were highly overlapped and were distributed in the hand area of SI in an orderly topography from thumb (lateral) to pinky fingers (medium). The distribution order was consistent with the homunculus organization of body parts in SI. A similar mirror pattern of SI activation topography was observed when electrically stimulating the contralateral fingers of amputee subjects. Results confirm that the evoked tactile sensation of fingers corroborates what was perceived by amputees. It is also consistent with previous finding using functional magnetic resonance imaging (fMRI) [43]. They recorded brain activities when mechanically stimulating the stump hand map area. They also demonstrated a somatotopic map of the phantom fingers in the hand region of SI. The corresponding cortical representation of sensory perception in SI during transcutaneous electrical nerve stimulation (TENS) of the upper stump was generally observed with EEG recordings [13]. When directly stimulating the median nerve and the unlar nerve with implanted transverse intrafascicular multichannel electrodes at the upper arm level, sensations perceived in different parts of the phantom hand corresponded to different evoked responses in the somatosensory cortex [44]. In our study, MEG results depicted both time response and response area of the SI activities with non-invasive stimulation of all five projected finger map (PFM) areas in the forearm stump. This confirmed that a direct neural connection exists between the peripheral PFM skin receptors and central SI neurons, similar as that of the intact sensory pathway in the contralateral hand. Furthermore, the central SI responses was correlated to the strength of peripheral electrical stimulation (Fig. 3). Thus, the feeling of lost fingers reported by amputees is caused by stimulating sensory fibers that innervate the fingers before amputation. The findings substantiated that the regenerated sensory afferent pathway can form a non-invasive neural interface capable of conveying finger-specific sensory information from prosthetic hands to the brain of amputees.

However, this approach may be limited to those amputees who developed naturally the evoked tactile sensation after amputation. At amputation, the severed nerve fibers are usually treated to prevent forming a neuroma at the ending [45]. It is commonly believed that the formation of neuroma is one of the causes for phantom pain in many amputees [46], [47]. However, our subjects with ETS reported that they experienced little or no phantom pain post amputation. Furthermore, before they were tested by our study, they rarely noticed ETS phenomenon existed in their stump skin. This seems to hint that new receptors grow into the stump skin from the severed sensory nerve, but at a deeper layer. Indeed, we had to poke the stump skin area of all subjects in order to elicit the feeling of lost fingers when defining the projected finger map (PFM). Electrical stimulation of PFM areas can always elicit the feeling of lost fingers, because electrical current can penetrate deeply into the skin and activate the newly grown sensory nerve fibers. It is not clear why only a fraction of amputee subjects exhibits such naturally formed PFM. But it may be possible to regrow the sensory nerve fibers onto the stump skin using the technique of targeted sensory re-innervation (TSR) [48] for those amputees without naturally formed PFM. A fMRI study showed that SI maps of targeted muscle and sensory reinnervation (TMSR) patients with upper level amputation displayed a greater similarity to normal ones, as compared to those of non-targeted muscle and sensory reinnervation patients [49]. Thus, TSR may be performed to enlarge the population of amputees who may benefit the technique of ETS based sensory feedback.

An interesting result observed in our subjects tested was that the topographies of SI responses with ETS were similar to those in the contralateral SI responses evoked from stimulating contralateral fingers. This observation corroborated the findings of other studies on plastic changes in sensory cortex after amputation [50]. They found that the somatotopic organization of sensory cortex are stable following amputation, and the frequently noticed hand/finger feelings on the face of amputees may be due to short-term potentiation of sub-cortical neurons. Our amputee subjects experienced little or no such mis-located feelings of hand/finger, and little or no phantom limb sensation (or phantom pain). Nevertheless, the stable somatotopic representation of hand/fingers in the SI cortex after amputation provides an important neural basis for homologous sensory integration between prosthetic hands and amputees. In this sense, ETS based sensory feedback developed in this study can greatly facilitate sensory integration with finger specificity between prosthetic hands and amputees.

This study reaffirmed the feasibility to restore a finger-to-finger sensory feedback for amputees with simultaneous multi-channel stimulation. In one experiment, the subjects were asked to press force sensors using the contralateral homonymous fingers when pressure was sensed in a prosthetic finger. This required that the subject first senses the pressure applied to a prosthetic finger, correctly identifies which prosthetic finger was pressed, and then instructs the same contralateral finger to press the force sensor with a pressure proportional to that applied to the prosthetic finger. This test illustrated the seamless sensorimotor information flow from prosthetic fingers to contralateral motor neurons, with a cognitive process to translate the incoming sensory information into a motor output of action. Results indicated that not only the accuracy of finger identification was higher than chance probability (20%) of all fingers in the three subjects, but also there was a trend of positive correlation between the prosthetic pressure and the contralateral pressure (Fig. 4). Nonetheless, the average response time of correct finger identification was not statistically different from that of incorrect finger identification in both Subject 2 and Subject 4, which was on the order of 2.5 (s). A short sensory-to-motor response time is essential for closed-loop control of prosthetic hand operation using sensory feedback via ETS. Yet, closed-loop implementation of this technique must also consider the potential disturbing effects of electrical stimulation to EMG recordings [51], [52].

## IV. Conclusion

This paper describes an approach to restitution of finger-specific sensory feedback in transradial amputees based on the phenomenon of evoked tactile sensation. In this study, we used the neuroimaging technique of Magnetoencephalography (MEG) to illuminate the direct neural pathway between somatosensory cortex (SI) and the stump skin that transmits finger-specific information of the amputated hand. Here we report the MEG findings of SI responses obtained during stimulating the projected finger map (PFM) on the stump skin in transradial amputees. The time course of evoked SI response followed the stimulus with a delay accountable to neural transmission from the peripheral stump to the SI cortex. The active regions of evoked response in the SI of amputated side were consistent to the finger/hand map of homunculus, which also exhibited a mirror image in the SI of the contralateral normal hand. There was a causal relationship between the strength of PFM stimulation and the intensity of SI activities, which manifested a direct connection from the PFM skin receptors to SI neurons. Test results indicated that the amputees can recognize a touch or pressure at the prosthetic fingers as if the corresponding missing finger was touched with the restored sensory information pathway. These findings confirm that a finger specific sensory ability of amputees can be restored via ETS. The reestablished sensory feedback pathway affords a unique non-invasive neural interface capable of communicating haptic information experienced at the prosthetic fingers back to the brain of amputees in a finger-to-finger specific way.

## Supplementary Materials

Supplementary materials contain the complete Methods employed in this study and two video files.

Video1_Finger identification via electrical stimulation of ETS on PFM

Video2_Demonstration of the finger-to-finger identification experiment


