# Supplementary material for: Restoring Finger-Specific Sensory Feedback for Transradial Amputees via Non-Invasive Evoked Tactile Sensation
Source: IEEE Open J Eng Med Biol. 2020 Mar 19;1:98–107. doi: 10.1109/OJEMB.2020.2981566 (PMC8979634; doi:10.1109/OJEMB.2020.2981566)
Supplement: Supplementary file 1 [file supp1-2981566.pdf]

Supplementary Materials

Restoring Finger-Specific Sensory Feedback for Transradial Amputees via Non-Invasive Evoked Tactile Sensation

Manzhao Hao, Chih-Hong Chou, Jie Zhang, Fei Yang, Chunyan Cao, Pengyu Yin, Wenyuan Liang, Chuanxin M. Niu, *Member, IEEE*, and Ning Lan*, *Senior Member, IEEE*

I. Methods

A. Subjects and ethics statement

Four transradial subjects with five projected finger regions in their stumps were recruited in this study (Subject 1, 51 years old, female, left wrist disarticulation; Subject 2, 54 years old, male, left transradial amputation; Subject 3, 45 years old, male, right transradial amputation; and Subject 4, 65 years old, male, left wrist disarticulation). All subjects had normal neurological and psychological functions and healthy condition to accomplish experiments. The subjects were explained with experimental procedures and signed the consent form before experiments. The Ethics Committee of Animal and Human Subject Studies of School of Biomedical Engineering, Shanghai Jiao Tong University approved this study. Subject 1 dropped out in the middle of study.

B. Identification of the PFM on the stump

The perception of lost fingers can be elicited in the forearm stump of selected amputees by poking specific stump regions mechanically or by electrical stimulation. We poked the subject's stump skin using a blunt pen with a 2 mm tip diameter to identify the projected finger map (PFM) on the stump skin. The subject could report the sensation of lost fingers, sometimes as clear as the digits of the fingers. The region of sensitive skin with poking and the most sensitive point (MSP) of each projected finger region were marked with color pen. For further confirmation, electrical stimulation was applied on the MSPs of each projected finger region to make sure that the subject could feel the evoked tactile sensation (ETS) at the lost finger.

The distribution of PFM regions at the stump of all subjects follows the similar order. For the subjects with amputation below the elbow, the PFM regions are mostly located in the palm side of the stump area, and the sensation corresponds to the palm side of the missing hand. The thumb PFM region is usually close to the radial side of the forearm and the pinky PFM region close to the ulnar side. Poking the thumb and pinky PFM regions sometimes may evoke the feelings of dorsal side of the fingers. Some subjects can have sensation of different digits of lost fingers (see Figures 1 & 2 in the main body of the paper). In this study, we choose the PFM regions with most sensitive point for electrical stimulation, which usually corresponds to the first digit (or tip) of the fingers.

C. Evoked MEG recordings

The neuroimaging technique of MEG was employed to reveal the central activities in the somatosensory (SI) cortex while electrically stimulating the projected finger regions of stump, as well as the fingers of the contralateral hand. The experiments were conducted with a 102-channal whole head MEG machine (Elekta, Helsinki, Finland) and a programmable stimulator (Master-9, Iso-Flex, A.M.P.I. Company, Israel). Surface stimulation electrodes used in MEG recording were non-woven surface electrodes (Yancheng Dalun Medical Equipment Co. Ltd, China). The reference electrodes were 2.5 (cm) in diameter. The stimulation electrodes were 2.5 (cm) in diameter for Subject 1 and then were cut to 1 (cm) in diameter for Subject 2 and Subject 3. The stimulation was a train of bi-phasic current pulses at 1 Hz. The pulse width was fixed at 200 us. The amplitude of stimulation current was varied as needed. When stimulating the fingers of contralateral hand, the subjects reported a feeling of knocking on the fingers. When the stimulation current was applied to the projected finger region on the stump, the subject described the feeling of tapping or knocking in the lost finger. The finger regions of PFM and the fingers of contralateral hand were stimulated with the 1Hz pulse train of 120 pulses in each trial. During the whole experiment, the subject was asked to close their eyes, relax, lie down and keep her/his body still in the MEG shield room. The whole brain activities were recorded by the MEG head scan system. The sampling rate was 1000 Hz. The raw MEG data were bandpass filtered with 0.03 to 330 Hz cutoff frequencies. The trigger signal of each pulse generated by the stimulator was recorded simultaneously by the MEG system for data synchronization.

D. MRI head model

After recording MEG trials, the brain imaging of the subject was obtained with the Magnetic Resonance Imaging (MRI) scanner (3.0 T, General Electric). The Freesurfer software [1] was used to process the MRI data and to reconstruct the brain model. The brain model included an MRI volume, the envelope of the cortex and the brain surface of the head. The Brainstorm software [2] was used to map the MEG activities onto the MRI brain model.

E. Central activities of ETS in the SI

For computing the brain source activities recorded with the MEG signals, the MRI brain model and MEG data were loaded together to the Brainstorm software. Three reference points, i.e. nasion, left ear and right ear, defined in the MRI scan were selected to register the MEG sensors. The epochs of MEG data were extracted by the trigger signal of stimulation. There were 120 epochs of data in each trial. The time of the epoch was set

from pre- 100 (ms) to post- 500 (ms) based on the trigger maker of stimulus. The average of epoch data was used to estimate the source of central activities generated by peripheral stimulation. The cortex surface was chosen in Brainstorm as the source space. The method of minimum norm imaging in the Brainstorm was used to find the image of cortical current dipole density. The dipole's orientation was constrained to be 'normal to cortex'. The number of electric dipoles was set to 15000, corresponding to the number of vertexes representing the cortex envelope. The MEG data representing the magnetic flux thereafter was converted into values of current dipole density at 15000 points of the brain model at each time instant of sampling.

In this experiment, the somatosensory cortex was the area of interest. In the time domain of the MEG data, there was a peak of the magnetic flux in the parietal lobe with a latency time in the range of 50 to 70 (ms) post stimulation. This latency time was consistent with the conduction time for the sensory afferent signal passing from the hand/forearm to the brain. From the 500 images of the current source density map corresponding to 500 (ms), the images around the time of the magnetic flux peak were visually inspected to find a gross response area in the somatosensory cortex. Within the gross response area, there was a group of vertexes identified with values of current source density above a threshold value of 12 to 75 (pA·m). The threshold was consistent in PFM regions or the contralateral hand in a subject. Then the area including these vertexes was marked as the response area (RA) in the SI. The average cross current source densities in the RA during the epoch time was also available for analysis. The response time (RT) was defined as the peak time of the average current source densities in the RA. The corresponding peak value was defined as the index of 'Mean' response intensity within the RA. The area value of the RA was defined as the index 'Area' for evaluating response intensity. The vertex with the maximum value of current dipole density in the RA was defined as the maximum response point (MRP). The value of current dipole intensity at the MRP at the RT instant was defined as the index of 'Maximum' response intensity. The three indices were used collectively for evaluating central response intensity within the landscape of the RA in the SI.

Three experiments were conducted with MEG recordings. The first experiment was designed to identify the response area of the central activities in the SI while peripherally stimulating the PFMs or the contralateral fingers. The thumb and pinky finger of the PFM and the contralateral hand were stimulated with a current amplitude that was sufficient to generate a sensation without causing tingling pain. The second experiment was to delineate the distributions of SI activation elicited with stimulating all fingers of the PFM and the contralateral hand. In the third experiment, each PFM regions or fingers of contralateral hand were stimulated by 3 to 5 pulse trains with amplitudes varied from low to high levels (from 0.25 to 8 mA with 1 cm in diameter of stimulation electrode). The minimal amplitude was the threshold and the maximum amplitude was that at which the subject felt the strongest sensation at about 8 mA. 5 levels of current amplitude were obtained with equal division of the modulation range between the minimal and maximum values. Due to availability of amputee subjects, not all them participated in the three experiments.

MEG response times (RT) obtained in 3 transradial amputee subjects when stimulating their projected finger regions in the stump skin and contralateral fingers were listed in Table I.

F. Multi-channel sensory stimulation system based on ETS

A non-invasive tactile sensory feedback system has been developed to demonstrate the feasibility to provide sensory feedback for forearm amputees based on the technique of ETS. The tactile sensory feedback system consists of two subsystems. The first subsystem is a data acquisition unit and a digital signal processing unit. The subsystem contains pressure and other types of transducers and a digital signal processor (DSP). The second subsystem integrates a number of distributed stimulator units (DSU), each of which outputs one channel of biphasic, regulated current pulse. This sensory feedback system can support sampling of multiple channels of sensory information and encoding of the sensory information into multiple channels of electrical stimulation in real time. In the experiments, surface stimulation electrodes were non-woven surface electrodes (Yancheng Dalun Medical Equipment Co. Ltd, China). The reference electrodes were 2.5 (cm) in diameter and the stimulation electrodes were cut to 1 (cm) in diameter. A commercial prosthetic hand (Keshen, Shanghai, China) was equipped force sensors (FlexiForce™, Tekscan, Inc., MA, USA), one on each finger. The outputs of prosthetic sensors were sampled by the sensory feedback

Table I. Response Time Measured from Area of Interest in SI (ms)

	Thumb		Index		Medius		Ring		Pinky	
Subjects	A	C	A	C	A	C	A	C	A	C
S1	55 (n=1)	57.5±2.5 (n=2)							56.5±1.5 (n=2)	53.5±0.5 (n=2)
S2	55.3±8.2 (n=6)	62.2±12.6 (n=6)	69.5±1.6 (n=6)	72.2±3.5 (n=6)	65.6±5.9 (n=5)	71.8±2.0 (n=6)	70.0±2.1 (n=4)	70.3±12.5 (n=6)	69.2±1.2 (n=5)	71.0±2.1 (n=6)
S3	70 (n=1)	55 (n=1)	77 (n=1)	68 (n=1)	78 (n=1)	57 (n=1)	61 (n=1)	61 (n=1)	63 (n=1)	62 (n=1)
Mean	57.1±8.6 (n=8)	60.3±10.7 (n=9)	70.6±3.0 (n=7)	71.5±3.5 (n=7)	67.7±7.1 (n=6)	69.7±5.5 (n=7)	68.2±4.1 (n=5)	69.0±12.0 (n=7)	65.3±5.6 (n=8)	66.1±7.5 (n=9)

A=Amputated side, C=Contralateral side

system, and used to encode the parameters of stimulation current, i.e. amplitude or pulse width. Another five force sensors were mounted on a hand shaped plate, which was pressed by the contralateral fingers of the subject. The outputs of the five contralateral finger sensors were used to compare with those of the prosthetic sensors. The feedback system was tested for its fidelity to transmit finger-specific information (see Video 1 for demonstration).

G. *Finger to finger identification tests*

Four sensory modalities were mostly noticeable with the size of stimulation electrode used (1 cm in diameter) [3], i.e. touch, buzz, vibration and numbness. In the following experiments, the modality of buzz sensation was chosen to encode the prosthetic pressure because of its wide modulation range. Stimulation frequency of 50 Hz was used throughout the experiments based on previous studies [4]. The initial current amplitude was set at 1 (mA) with a fixed pulse width of 200 (us). Stimulation current was then incremented at 1 (mA) step size, until the subject felt an obvious buzz sensation. After the amplitude was determined, the pulse width was increased continuously starting from 0 (us) with a step size of 20 (us). Then the minimum and the maximum pulse widths for buzz sensation were obtained, denoted as w_{min} and w_{max}, respectively, for each fingers of PFM regions and contralateral hand. A linear model was designed for encoding prosthetic pressures. The stimulation pulse width (w) was modulated with the prosthetic pressure, p, as follows:

$$W = \begin{cases} 0, & p < p_{min} \\ \dfrac{W_{max} - W_{min}}{p_{max} - p_{min}} * (p - p_{min}) + W_{min}, & p_{min} \le p < p_{max} \\ W_{max}, & p \ge p_{max} \end{cases} \tag{1}$$

Where w is the pulse width of stimulation, p is the value of prosthetic pressure recoded, pmax is the maximum pressure set at 15 (N), and pmin is the minimum pressure set at 1.5 (N).

The finger to finger identification (FFI) was quantified in such a way. The prosthetic fingers were pressed by an experimenter, the subject was then asked to use his/her contralateral fingers to press the sensors of the homonymous finger felt with stimulation of the PFM regions at the stump. To familiarize the subject with experimental setting, a 5-minute pre-test was given to each subject, in which the 5 fingers of the prosthetic hand were pressed from thumb to pinky and from pinky to thumb, respectively, with the subject viewing the prosthetic hand. not for training.

In experiments, subject's view to the prosthetic hand was blocked. In each session, the five fingers of the prosthetic hand were pressed 4 times in a random order with a total of 20 trials. There were 6 sessions in one experiment with a total of 120 trials. A 5-minute break was allowed between two sessions. The subject was explicitly required to press the sensor by the contralateral finger with a force proportional to the perceived sensory strength of the pressure at the prosthetic finger (See Video 2 for demonstration).

In these trials, 10 channels of pressure data from the prosthetic sensors and the contralateral finger sensors were recorded for finger to finger identification analysis. The identification performed by the subject was also recorded by an experimenter. The confusion matrix was used to overview the identification performance of five fingers. The response time of an identification was calculated from the pressure data. In the case of Subject 3, due to sensor failure in the contralateral hand during experiment, pressure data were not used in later analysis.

II. References

[1] B. Fischl, "FreeSurfer," *NeuroImage*, vol. 62, no. 2, pp. 774–781, Aug. 2012.

[2] F. Tadel, S. Baillet, J. C. Mosher, D. Pantazis, and R. M. Leahy, "Brainstorm: a user-friendly application for MEG/EEG analysis," *Comput. Intell. Neurosci.*, vol. 2011, p. 879716, 2011.

[3] P. Li, G. Chai, K. Zhu, N. Lan, and X. Sui, "Effects of electrode size and spacing on sensory modalities in the phantom thumb perception area for the forearm amputees.," *Conf. Proc. Annu. Int. Conf. IEEE Eng. Med. Biol. Soc. IEEE Eng. Med. Biol. Soc. Annu. Conf.*, vol. 2015, pp. 3383–3386, 2015.

[4] G. Chai, X. Sui, S. Li, L. He, and N. Lan, "Characterization of evoked tactile sensation in forearm amputees with transcutaneous electrical nerve stimulation," *J. Neural Eng.*, vol. 12, no. 6, p. 066002, Dec. 2015.
